# The first juvenile specimen of *Manchurochelys manchoukuoensis* from the Early Cretaceous Jehol Biota

**DOI:** 10.7717/peerj.3274

**Published:** 2017-04-26

**Authors:** Shuai Shao, Yang Yang, Lan Li, Da-Yong Sun, Chang-Fu Zhou

**Affiliations:** 1 Research Center of Palaeontology & Stratigraphy, Jilin University, Changchun, Jilin, China; 2 Paleontological Institute, Shenyang Normal University, Shenyang, Liaoning, China; 3 School of Earth and Space Sciences, Peking University, Beijing, China; 4 College of Earth Science and Engineering, Shandong University of Science and Technology, Qingdao, Shandong, China

**Keywords:** Early Cretaceous, Jehol Biota, Sinemydidae, Juvenile features, Ontogeny, Diversity

## Abstract

A small juvenile turtle is described from the Early Cretaceous Jehol Biota, shedding light on the juvenile morphology and ontogeny of *Manchurochelys manchoukuoensis*. Several juvenile features are uncovered, such as a small and circular carapace (less than half of the adult), wide vertebral scales, and lateral carapacial fontanelles. In contrast to the adult morphology, which has an oval carapace, closed lateral fontanelles, and longer vertebrals 2–4, the juvenile of *M. manchoukuoensis* is more comparable to that of *Sinemys lens*, except for earlier occurrence of the well-ossified carapace of the latter. Differs from *Changmachelys bohlini*, and *Ordosemys liaoxiensis*, in which the circular carapace is relatively independent of ontogenetic age, and the lateral fontanelles are only closed in adult stage of *O. liaoxiensis*. Therefore, the trajectory of ontogenetic change appears to be highly diversified in the sinemydids.

## Introduction

As part of the basal eucryptodires, the sinemydid turtles are dominated and widely distributed in the Early Cretaceous of East Asia (e.g., Hirayama, Brinkman & Danilov, 2000; Rabi, Joyce & Wings, 2010; Joyce et al., 2016). In the last decades, sinemydids (*Manchurochelys manchoukuoensis*, *Ordosemys liaoxiensis*, *Liaochelys jianchangensis*, *Xiaochelys ningchengensis*) have been discovered from the Jehol Biota and a large number of well-preserved fossils have been described (e.g., Tong, Ji & Ji, 2004; Zhou, 2010a; Zhou, 2010b; Zhou & Rabi, 2015). However, descriptions are mainly focused on the taxonomic diversity, with little focus on ontogenetic change. To date, three ontogenetic series have been reported in the Sinemydidae. As known in *Sinemys lens* (Brinkman & Peng, 1993), the ontogenetic series show the morphology varied from the juvenile to adult, with the carapacial outline is nearly rounded in the juvenile and becomes to an oval in an adult; vertebral scales 2–4 are wide in juveniles but much narrower in adults. In contrast, the ontogenetic change is relatively less developed in *O. liaoxiensis* (Tong, Ji & Ji, 2004) and *Changmachelys bohlini* (Brinkman et al., 2013).

Here, a juvenile turtle from Yixian Formation of Yixian, western Liaoning, which is the same as the type locality and horizon of *Manchurochelys manchoukuoensis* (Endo & Shikama, 1942; Zhou, 2010b), is described. It is similar to *M*. *manchoukuoensis* in cranial and shell morphology, sharing features such as the postorbital isolated from the squamosal, a relatively elongated crista supraoccipitalis, and a smaller anterior suprapygal. In addition, the juvenile features, such as a nearly circular shell, open lateral fontanelles and wider vertebral scales, are shown for the first time in *M*. *manchoukuoensis* by this specimen. This specimen shows that the ontogeny of *M*. *manchoukuoensis* is more comparable to that of *S. lens*, than that of *C. bohlini* or *O. liaoxiensis*.

## Material and Methods

**Figure 1:**
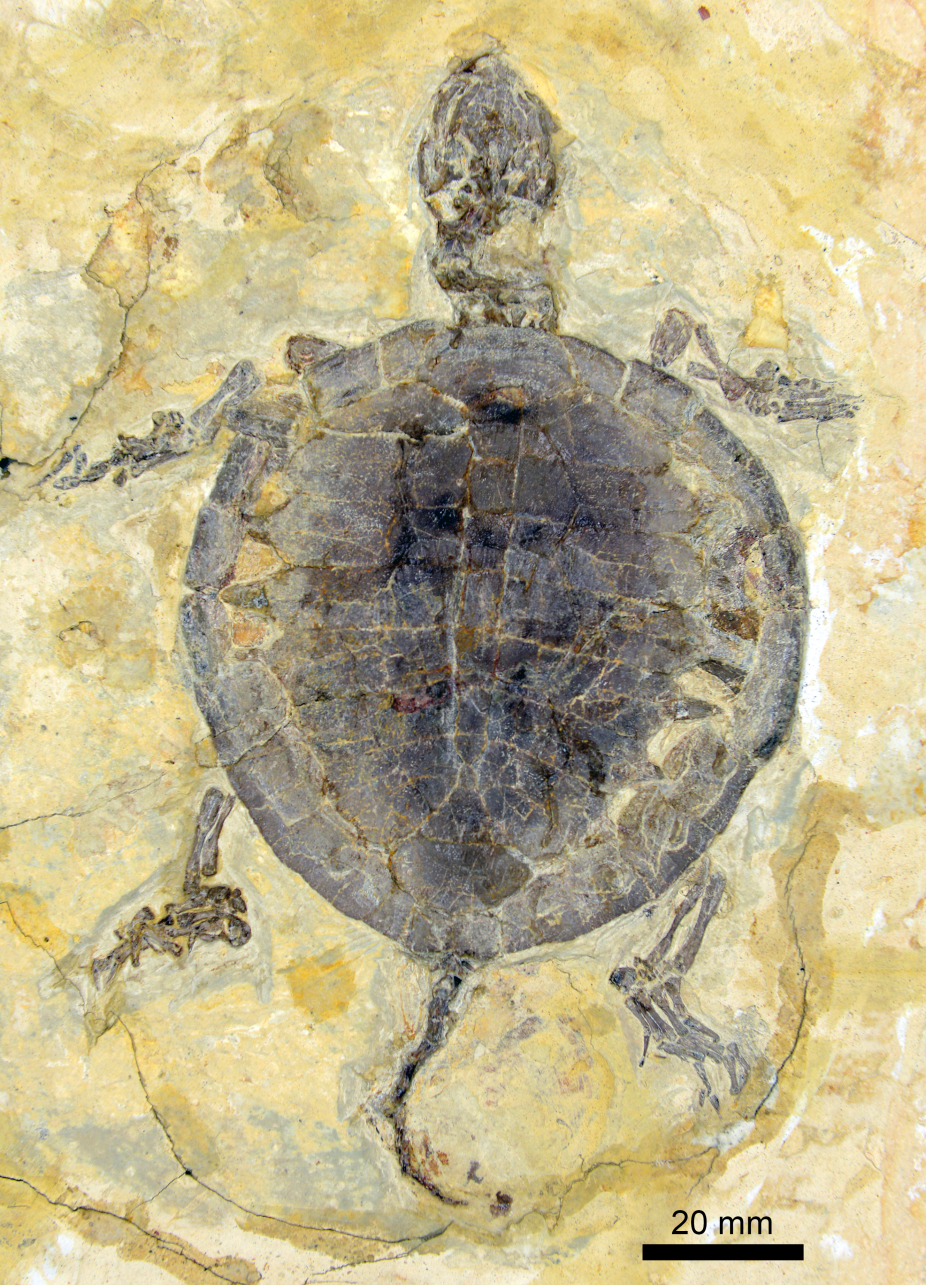
Juvenile specimen of *Manchurochelys manchoukuoensis* (PMOL-AR00007).

**Figure 2:**
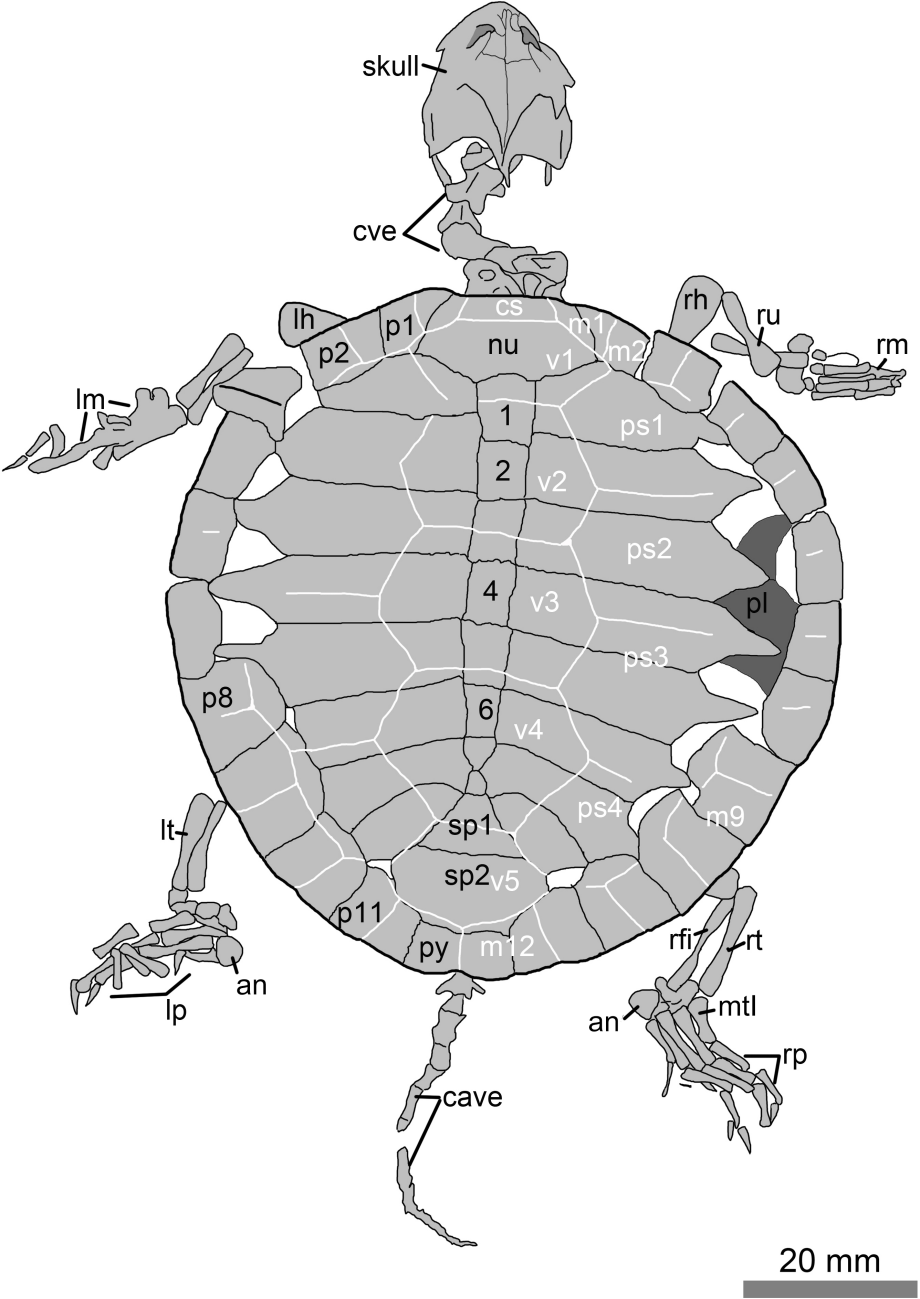
Line drawings of the juvenile specimen of *Manchurochelys manchoukuoensis* (PMOL-AR00007). Abbreviations: an, ansulate bone; c1–c8, costal plates 1–8; cave, caudal vertebrae; cs, cervical scale; cve, cervical vertebrae; lh, left humerus; lm, left manus; lp, left pes; lt, left tibia; m1–12, marginal scales 1–12; mtI, metatarsal I; nu, nuchal; p1–p11, peripheral plates 1–11; pl, plastron; ps1–4, pleural scales 1–4; py, pygal; rfi, right fibula; rh, right humerus; rm, right manus; rp, right pes; rt, right tibia; ru, right ulna; sp1–sp2, suprapygals 1–2; v1–v5, vertebral scales 1–5; 1–8, neural plates 1–8.

**Figure 3:**
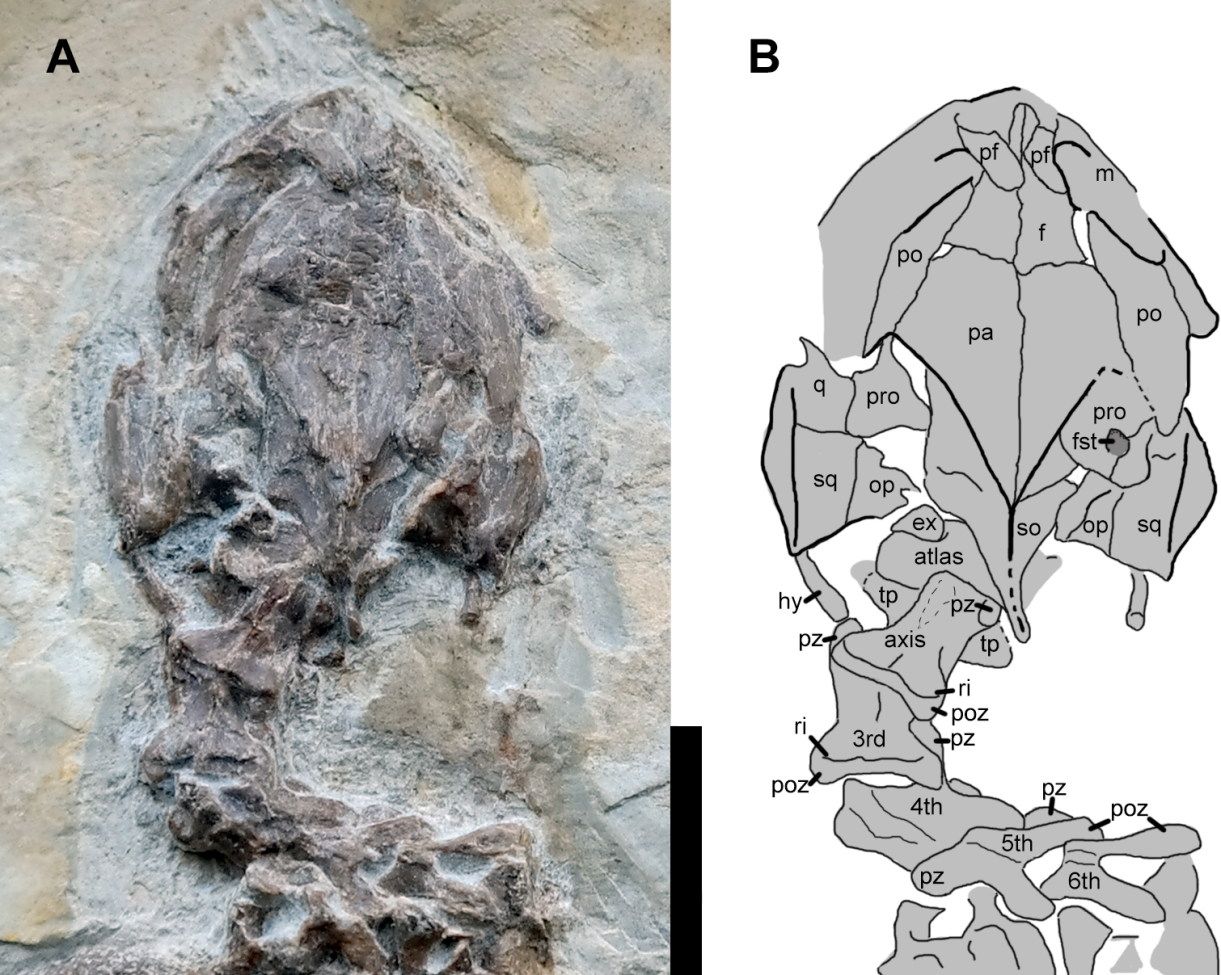
Cranium and cervical vertebrae of the juvenile specimen of *Manchurochelys manchoukuoensis*. (PMOL-AR00007). Abbreviations: ex, exoccipital; f, frontal; fst, foramen stapedio-temporale; hy, hyoid; m, maxilla; op, opisthotic; pa, parietal; pf, prefrontal; po, postorbital; poz, postzygapophysis; pro, prootic; pz, prezygapophysis; q, quadrate; ri, ridge; so, supraoccipital; sq, squamosal; tp, transverse process of the cervical vertebra; 3rd–6th, cervical vertebrae 3–6. Scale bar = 10 mm.

As in other turtles of the Jehol Biota, the fossil (PMOL-AR00007; Figs. 1–3) is strongly compressed so is preserved two dimensionally. After the professional preparation in Paleontological Museum of Liaoning (PMOL), the fossil was well exposed in dorsal view. It is a nearly complete skeleton, including skull, cervical and caudal series, carapace, forelimbs and hindlimbs. These elements are well stretched out of the carapace. The plastron is a little exposed through the lateral fontanelles of the carapace. As a juvenile individual, the specimen is small in size, with a carapace length of 78.5 mm, less than half of the adult (PMOL-AR00008). The lateral carapacial fontanelles between the costals and peripherals are open.

## Description and Comparison

### Skull

The skull is subtriangular in dorsal view (Figs. 1–3). The preorbital portion is distinctly narrower, relative to the broad postorbital portion. However, the preorbital portion is poorly defined. On the interorbital roof, the prefrontal is slender and more extensive medially, and seems to have a little area of contact with its counterpart, as in *Manchurochelys manchoukuoensis* (PMOL-AR00008 and PMOL-AR00180; Zhou, 2010b; Zhou, Rabi & Joyce, 2014). The midline contact of the prefrontals is widely present in the sinemydids, except for *Ordosemys* spp. and *Sinemys* spp., in which the prefrontals are fully separated by the frontals. The frontal is elongate and bears a slender anterior process. The process is exposed in front of the prefrontal midline contact. A similar condition is possibly present in *Liaochelys jianchangensis*, although the anterior process of the frontal was identified as the nasal (Zhou, 2010a). The main body of the frontal is wide laterally, and has a broad contact with the postorbital. Between the prefrontal and postorbital, the frontal contributes to orbit margin. Posteriorly, the parietal is a massive element, and forms the main part of the cranial roof. Anterolaterally, the parietal contacts the postorbital along a broad suture. Posterolaterally, the parietal, together with the postorbital, borders the upper temporal emargination. The upper temporal emargination is deep and fully exposes the processus trochlearis oticum in dorsal view. Between the temporal emarginations, the parietals are narrow and contact the crista supraoccipitalis distally. As in PMOL-AR00008 and PMOL-AR00180 (Zhou, 2010b; Zhou, Rabi & Joyce, 2014), the crista supraoccipitalis is relatively elongated, extending well beyond the level of the squamosal crests. The postorbital is isolated distally, as in *M*. *manchoukuoensis* (PMOL-AR00008 and PMOL-AR00180; Zhou, 2010b; Zhou, Rabi & Joyce, 2014), and *Sinemys* spp. The squamosal is positioned at the posterolateral corner of the skull, and develops a low crest that borders the lateral boundary of the upper temporal emargination. Within the temporal emargination, the foramen stapediotemporale is large and enclosed by the prootic and quadrate.

### Shell

The low domed carapace is nearly circular, with a length of 78.5 mm. Its surface is relatively smooth, but distinctly sculptured by the scale sulci and plications. The lateral fontanelles are open along the medial side of the peripherals, in contrast with the closed condition in the adults (the lost holotype and PMOL-AR00008; Endo & Shikama, 1942; Zhou, 2010b). Through the fontanelles, the right hyoplastron and hypoplastron of the plastron are partially exposed. They have a nearly straight lateral margin, possibly implying absence of the lateral fenestra.

Along the midline of the carapace, the nuchal is a large element with a trapezoid-like outline, different from a rectangular condition in PMOL-AR00008 (Zhou, 2010b). It is roughly twice as wide as it is long. The anterior margin of the nuchal is slightly convex, as in PMOL-AR00180, but in contrast with slightly concave condition in PMOL-AR00008 (Zhou, 2010b). However, this convexity is less than the anterior expansion of the first peripherals. Therefore, a shallow emargination is present along the anterior margin of the nuchal and bordered on either side by the first peripherals. The nuchal widens posteriorly and has a curved posterior margin, as in PMOL-AR00180 (Zhou, Rabi & Joyce, 2014), but in contrast to PMOL-AR00008, in which the anterior and posterior margins are nearly parallel and comparable in width (Zhou, 2010b).

Behind the nuchal bone, eight neural bones are arranged in a row. Neurals 1–6 are rectangular, and gradually reducing their size posteriorly along the row. In the adult specimen (PMOL-AR00008), however, the neurals are varied in shape and size (Zhou, 2010b). The last two neurals 7–8 become subtriangular and strongly reduced. They build a narrow connection, different from a relatively broad contact in PMOL-AR00008 (Zhou, 2010b). The eighth neural is completely separated from the eighth costals by the seventh costals. In contrast, the eighth neural is only connected with the eighth costal plates in PMOL-AR00008 (Zhou, 2010b).

Following the neural row, two suprapygals are developed. The first suprapygal is distinctly smaller than the second one. However, it is proportionately smaller than the suprapygal I of PMOL-AR00008 (Zhou, 2010b). The pygal is the last element along the midline of the carapace. It is wider than long, and bears a shallow emargination at the midpoint of its posterior margin.

As a juvenile specimen, the costal plates are not fully ossified at their distal ends. Therefore, the lateral fontanelles are present between the costals and peripherals. In contrast, the fontanelles are closed in the adults (the lost holotype and PMOL-AR00008; Endo & Shikama, 1942; Zhou, 2010b). Proximally, the costals are firmly articulated with the adjacent elements.

Peripherals, along with the nuchal and pygal, form the outline of the carapace. Anterior peripherals are guttered by an upturned edge. The first peripheral is sub-triangular, and has a broad contact with the first costal posteriorly. The peripheral extends anteriorly, slightly beyond the anterior margin of the nuchal. Therefore, the first peripherals border the shallow nuchal emargination. The last four peripherals are well expanded medially, and nearly closed the fontanelles with the corresponding costals in the left side; while the fontanelles are still open in the right side.

Scale configuration is identifiable through the marked sulcus. Plications are weakly developed along the transversely oriented sulci of the vertebral and pleural scales. The plications extend posteromedially on the vertebral scales, whereas the plications on the pleural scales are posterolaterally extended. The cervical scale is small and wider than long. The vertebral scales are hexagonal and distinctly wider than long, in contrast with the adults (the lost holotype and PMOL-AR00008), in which the vertebrals 2–4 are longer than wide (Endo & Shikama, 1942; Zhou, 2010b). The first vertebral is shorter than the others. It has a longer contact with the first marginal than with the second marginal, which is different from the condition in PMOL-AR00008 and PMOL-AR00180. It is wider than the nuchal plate, and partially covers the first peripherals, first costals and first neural. The second vertebral is much longer than the first one, but shorter than the vertebrals 3–4. Vertebral 2 is not regular hexagonal in having the shorter anterior margin and longer anterolateral margins. Vertebral 3 is a large element, but slightly shorter than vertebral 4. The sulcus of vertebrals 3–4 crosses the posterior part of the fifth neural, as in other sinemydids. Vertebral 4 is reduced posteriorly with a shorter posterior margin. Also, its posterolateral margins are distinctly longer than the anterolateral margins. The sulcus of vertebrals 4–5 crosses the middle portion of the first suprapygal. In the adult (PMOL-AR00008), the sulcus crosses the suture between the eighth neural and the first suprapygal (Zhou, 2010b). The last vertebral is the narrowest, and is subequal with the second suprapygal in width. Posteriorly, the sulci of vertebral 5 and adjacent marginals are limited to the suprapygal. Most of pleural scales are not certain in outline since they lack the continuous sulci with the marginals scales. On the left side, pleural 3 appears to be as wide as it is long, which is different from the wider condition in adult. In contrast, the outline of pleural 4 is comparable to that of the adult in proportion. The marginal scales are also somewhat uncertain. The first marginal is small and subtriangular with a broad contact with cervical and the first vertebral. Marginals 2–3 are subrectangular. The last five marginal scales 8–12 are distinctly enlarged. These are well limited on the peripherals. Marginal 12 is the exception and slightly extends onto the second suprapygal.

### Cervical series

The articulated cervicals form a S-shaped cervical series (Figs. 1– 3). The second to sixth cervicals are well exposed in dorsal view. The atlas is represented by a displaced neural arch. It is partially exposed without valuable information. The axis is anteriorly positioned against the crista supraoccipitalis, and articulates the third cervical posteriorly. Its neural spine is broken off, except for a low portion along the posterior third of the neural arch. The neural arch is constricted at the middle portion, forming a dumbbell-shaped outline. At the anterolateral corner, the prezygapophysis is slightly developed with a plate-like facet. The facet is dorsolaterally directed, in contrast to the dorsomedially-directed condition in the succeeding cervicals. Posteriorly, the postzygapophyses are expanded more laterally than posteriorly, forming a nearly straight posterior margin to the neural arch, as in PMOL-AR00180. Between the postzygapophyses, a shelf-like ridge is developed parallel to the posterior margin of the neural arch. Anterior to the ridge, a shallow concavity is present on either side of the low neural spine. The transverse process is well developed along the anterior half of the axis (Fig. 3).

The third cervical is relatively shorter than the axis. The neural spine of the third cervical is also broken off. Based on its remains, the neural spine is relatively reduced, and only present on the anterior half of the neural arch. As in the axis, the shelf-like ridge is developed which is parallel to the posterior margin of the neural arch. In the fourth cervical, the left side is somewhat damaged and the left prezygapophysis and postzygapophysis are broken off. The neural spine is low. The posterior margin of the neural arch is V-shaped as a result of the deeply divergent postzygapophyses. The V-shaped posterior margin is highlighted in the fifth and sixth cervicals (Fig. 3). Due to the preservation, the last two cervicals are obscured.

### Caudal series

Seven caudal vertebrae are preserved in articulation extending from the carapace. The posterior half of the first caudal is exposed. Its anterior part is still overlapped by the carapace. The transverse process extends more laterally than posteriorly. The second caudal is slightly displaced from its original position, and exposed in posterior view. The posterior articular facet of the centrum is a shallow concavity, implying a presence of an opisthocoelous or amphicoelous centrum. The neural canal is relatively smaller than the centrum. The third caudal is wholly exposed in dorsal view, although the posterior part of the neural arch is broken off. The prezygapophyses are nearly paralleled to each other. The articular facet is strongly oblique. The transverse process is positioned more posteriorly than on the first caudal. The process extends more posteriorly than laterally, and well beyond the level of the vertebral body. The transverse processes reduce their size gradually in the succeeding caudals, and appear to be absent in the sixth or seventh caudals. The neural spine is well preserved in the sixth caudal. The spine is low, broad and rounded.

Another segment of the caudal series includes 12 vertebrae, possibly representing the distal caudals. This segment is displaced from the anterior portion. The first vertebra of the segment is strongly reduced, and is roughly half the length of the sixth caudal of the anterior portion. The complete number of caudals is therefore uncertain now. These caudals are also more or less damaged. They are exposed in lateral view, and have a distinctly curved ventral margin.

### Appendicular skeleton

In the forelimb, humerus bears an expanded distal end. The ectepicondyle foramen is open. The ulna and radius are slender and rod-like. They are subequal with a length of 10.4 mm. The ulna is relatively stronger than the radius. The manus is poorly preserved on both sides.

The hindlimb is well preserved on the right side. The tibia and fibula are about 13.4 mm long. The tarsals are positioned between the tibia and fibula and metatarsus. However, a further identification of the tarsal elements is difficult. Metatarsals I-IV are rod-like. The metatarsal I is stout with a length of 5.7 mm. The metatarsals II–IV are slender and long. Of these, the metatarsal III with a length of 8.1 mm, is the longest. The ansulate bone is large and hook-like. The pedal formula is 2-3-3-3-3. The digits gradually elongate from the digit I to reach the maximum length (16.2 mm) in the digit III, and then digits IV and V become shorter. In contrast, the digit V is 12 mm long and much more slender than other digits. In each digit, the phalanges decrease distally in size. The claw is well developed in anterior four digits. However, the ultimate phalanx is needle-like in the digit V.

## Discussion

### Affinity of the juvenile specimen (PMOL-AR00007)

Based on the above description, PMOL-AR00007 is a juvenile characterized by a small size (78.5 mm in carapace length), a circular carapace, lateral carapacial fontanelles, and wide vertebral scales. Few juveniles of sinemydids are known, except for *Sinemys lens*, *Changmachelys bohlini* and *Ordosemys liaoxiensis*. In contrast to PMOL-AR00007, the juvenile carapace of *S*. *lens* is well developed even in the smallest individual (less than 50 mm in carapace length), while the carapace of *C*. *bohlini* retains large lateral fontanelles throughout the whole growth series (Brinkman & Peng, 1993; Brinkman et al., 2013). Therefore, PMOL-AR00007 is confidently excluded from *S*. *lens* and *C*. *bohlini*. Also, PMOL-AR00007 differs from *O*. *liaoxiensis*, in which the smallest carapace is 120 mm long with the larger lateral fontanelles and wide vertebral scales.

Besides these ontogenetic features, PMOL-AR00007 shows a high similarity with *Manchurochelys manchoukuoensis* in having a short midline contact of the prefrontals, a postorbital-squamosal separation, a relatively elongated crista supraoccipitalis, and a smaller anterior suprapygal. Also, the specimen is excluded from *Sinemys* spp. in having a medial prefrontal contact, an elongate crista supraoccipitalis, presence of a cervical scale, a pygal, and eight neurals, and absence of the lateral spine on peripheral 7; *Dracochelys bicuspis* in having a short medial contact of the prefrontals, a shallow nuchal emargination, eight neurals, a contact of the first peripheral and the first costal, and upturned peripherals. Moreover, the fossil differs from *Ordosemys* spp., *Liaochelys jianchangensis, Xiaochelys ningchengensis, C. bohlini, Kirgizemys* spp., *Judithemys sukhanovi*, and *Macrobaena mongolica* in not having a postorbital-squamosal contact. In particular, the specimen is distinct from *Ordosemys* spp. in lacking a preneural plate, but having a long supraoccipital crest, a postorbital-squamosal separation, a broad contact of the first peripheral and the first costal, and a small first suprapygal; *L. jianchangensis* in having a long supraoccipital crest, and a postorbital-squamosal separation; *X. ningchengensis* in having a postorbital-squamosal separation, and a small first suprapygal; *C. bohlini*, *Kirgizemys* spp., *J. sukhanovi*, and *M. mongolica* lacking a broad medial contact of the prefrontals, broad interorbital roof (*M*. *mongolica*), and two subequal suprapygals (unknown in *C*. *bohlini*). Therefore, it is reasonable to assign the juvenile specimen (PMOL-AR00007) to *M*. *manchoukuoensis*.

### Ontogeny of *Manchurochelys manchoukuoensis*

**Figure 4:**
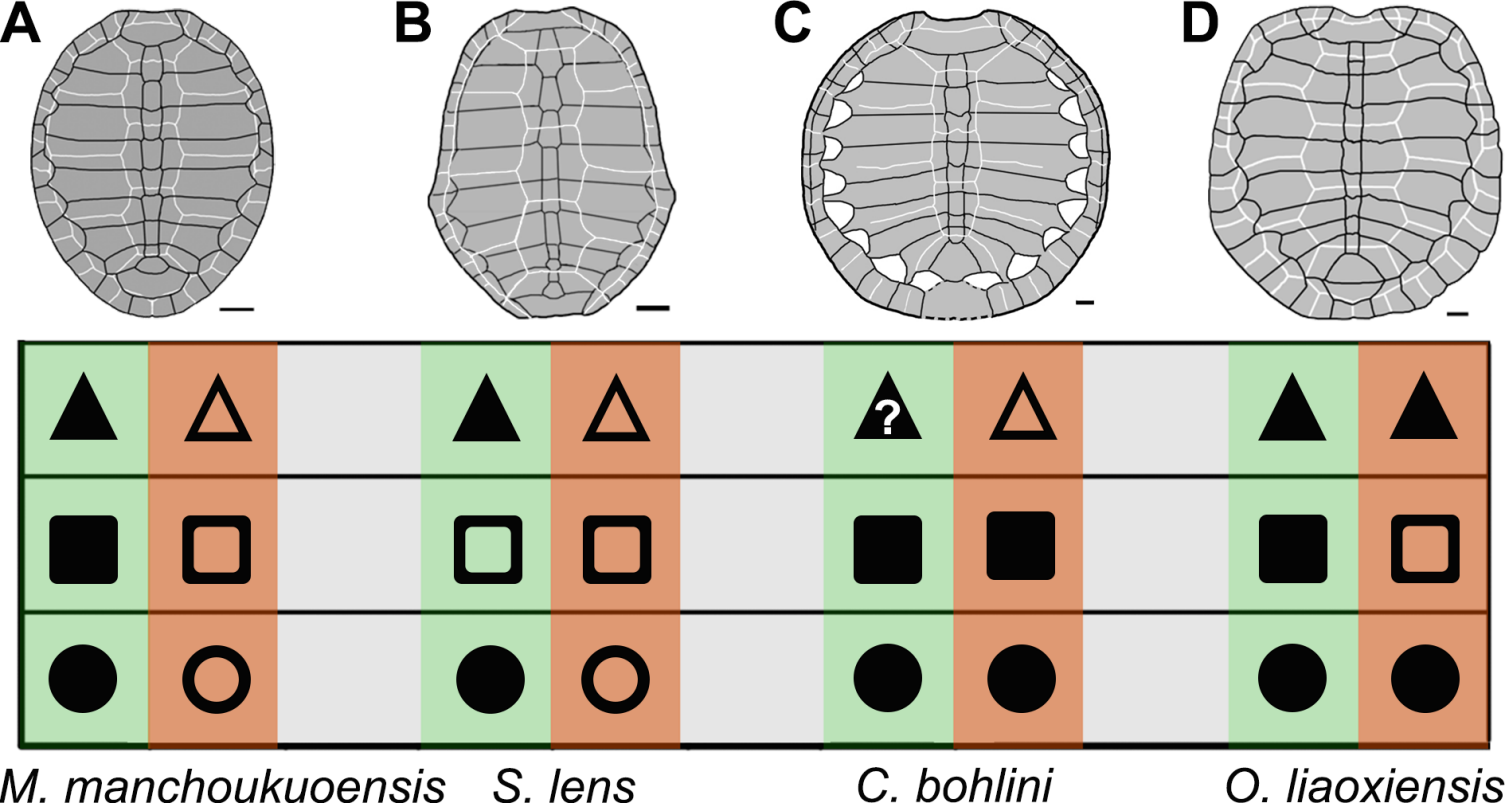
Comparison of ontogenetic patterns of sinemydids. Green column is juvenile stage, orange column is adult stage. Circle is shell outline: circular (solid circle); oval (empty circle). Square is lateral carapacial fontenalle: open (solid square); closed (empty square). Triangle is vertebral scales 2–4 in proportion: wider than long (solid triangle), longer than wide (empty triangle). Carapaces of *Manchurochelys manchoukuoensis* (A), *Sinemys lens* (B), *Changmachelys bohlini* (C), and *Ordosemys liaoxiensis* (D). Scale bars equal to 20 mm.

As the first juvenile individual, the specimen (PMOL-AR00007) provides an opportunity to understand the juvenile morphology and ontogeny of *M*. *manchoukuoensis*. Besides the small size, the juvenile features are remarkable in carapace morphology, such as a circular outline, open lateral fontanelles, and wider vertebral scales. In the adult specimen (PMOL-AR00008), the carapace is oval and longer than wide; the lateral fontanelles are completely closed in the well-ossified carapace; among the vertebral scales, vertebrals 2–4 are longer than wide (Fig. 4A, Zhou, 2010a). The similar condition is possibly present in the lost holotype (Endo & Shikama, 1942), which is similar to PMOL-AR00008 in size. Therefore, during growth of *M*. *manchoukuoensis*, proportions of the carapace has a positive allometry in length, resulting in a change from the circular to the oval outline. A similar ontogenetic pattern is also known in *Sinemys lens* (Brinkman & Peng, 1993). However, in *Changmachelys bohlini* and *Ordosemys liaoxiensis*, the proportion of the carapace of juveniles and adults is similar, so appears to be independent of the ontogenetic growth. Secondly, closure of the lateral carapacial fontanelles possibly occurs later in the ontogeny of *M*. *manchoukuoensis*. In contrast, the costo-peripheral fontanelles are closed in an earlier stage of *S*. *lens* (less than 50 mm in the carapace length; Brinkman & Peng, 1993), in a later stage of *O*. *liaoxiensis* (160 mm in the carapace length; Tong, Ji & Ji, 2004), but absent in *C*. *bohlini* (the largest carapace is 340 mm in length; Brinkman et al., 2013). Thirdly, as in *S*. *lens*, the vertebrals 2–4 show positive allometry in length relative to their width. However, in *O*. *liaoxiensis*, the vertebrals are wider than long, so proportions are independent of the ontogenetic stage. Based on these ontogenetic features, the ontogeny of *M*. *manchoukuoensis* appears to be more comparable to that of *S*. *lens*, than to *C*. *bohlini* and *O*. *liaoxiensis*, which is consistent with their closer phylogenetic relationship.

### Diversity in ontogenetic patterns of sinemydids

In the family Sinemydidae, the ontogenetic pattern is little known, but appears to be highly diversified (Fig. 4). The ontogeny is better known in *Sinemys lens*, the type species of the family Sinemydidae. All individuals of *S*. *lens* come from a single formation and greatly differ in size, and are interpreted to represent an ontogenetic series (Wiman, 1930; Ye, 1973; Brinkman & Peng, 1993). The smallest individual of *S*. *lens* has a circular, well-ossified carapace, wide vertebrals and a well-developed spine on peripheral 7 whereas in successively larger individuals the carapace becomes gradually more elongated with narrower vertebrals and a reduced peripheral spine. In large specimens, the carapace is oval, the verterbrals 2–4 longer than wide, and the peripheral 7 is corner-like, rather than spine-like (Fig. 4B; Wiman, 1930; Ye, 1973; Brinkman & Peng, 1993). In contrast, a different pattern is possibly present in another species of *Sinemys*, *S*. *gaffneyi*, since the unclosed lateral fontanelles and a large peripheral spine are present in a relatively larger individual (estimated carapace length is about 200 mm; Brinkman & Peng, 1993).

The ontogenetic series is also known in *Changmachelys bohlini* (Brinkman et al., 2013), and this shows a different ontogenetic history. Four individuals are greatly different in size are known, representing four stages from 47 mm to 340 mm in carapacial length. In contrast to *S. lens* and *Manchurochelys manchoukuoensis*, a circular shell is well known in these individuals and appears to be independent of the ontogenetic stages. In contrast, the carapace is poorly ossified with lateral fontanelles, even in the largest specimen, which is disarticulated somewhat (Fig. 4C, Brinkman et al., 2013). Unfortunately, the vertebral scales are only known in the largest carapace, hindering a further investigation in ontogeny. However, vertebrals 2–4 are longer than wide, as in the adults of *S. lens* and *M. manchoukuoensis*. Therefore, in the proportions of the vertebral scales, the possibility that *C*. *bohlini* may has a comparable ontogenetic change with the latter two cannot be excluded.

In *Ordosemys liaoxiensis*, juvenile and adult specimens are also known (Ji, 1995; Li & Liu, 1999; Tong, Ji & Ji, 2004), providing some information in growth series. They show a different trajectory of the ontogenetic change, compared to other sinemydids. These specimens vary from 120 mm to 213 mm in carapacial length. A well-ossified carapace is present in specimens ranging from 160 mm to 213 mm in carapacial length (Fig. 4D, Zhou, 2010a). In the smaller specimens, the carapace is poorly ossified with large lateral fontanelles. This condition seems to be comparable with that of *M*. *manchoukuoensis*, but different from *S. lens* and *C*. *bohlini*. As in *C*. *bohlini*, the carapace is circular and does not change during growth. The vertebral scales are all wider than long, so proportions of these scales are possibly independent of the ontogenetic stage. In contrast, the proportions of vertebral scales, especially vertebrals 2–4 change ontogenetically in other known sinemydids.

## Conclusion

Based on the juvenile specimen, our study firstly reveals the juvenile morphology and ontogeny of *Manchurochelys manchoukuoensis*, such as a circular carapace, wide vertebral scales, and lateral carapacial fontanelles, which distinct from the adult condition (carapace oval, lateral fontanelles closed, and vertebrals 2–4 longer than wide). The comparative study of *M*. *manchoukuoensis* and other sinemydids indicates the trajectory of ontogenetic change appears to be highly diversified in the sinemydids. Of these, the ontogenetic pattern of *M*. *manchoukuoensis* is more comparable to that of *Sinemys lens*, except for earlier occurrence of the well-ossified carapace of the latter. In *Changmachelys bohlini* and *Ordosemys liaoxiensis*, the different ontogenetic patterns are also known, such as the circular carapace is relatively independent of ontogenetic stage, and the lateral fontanelles are only closed in adult stage of *O*. *liaoxiensis*. In contrast to other taxa poorly-known in ontogenetic change, this diversification of the sinemydids is preliminary in our study and will be highlighted by future studies.

## Additional Information and Declarations

### Data Availability

The following information was supplied regarding data availability: The raw data is included in the figures in the manuscript.
